# Bcl-2抑制剂venetoclax在急性髓系白血病中的应用

**DOI:** 10.3760/cma.j.issn.0253-2727.2021.05.018

**Published:** 2021-05

**Authors:** 仪 张, 洁 金

**Affiliations:** 浙江大学附属第一医院血液科，杭州 310003 The First Affiliated Hospital of Zhejiang University, Hangzhou 310003, China

B细胞淋巴瘤2（B-cell lymphoma 2，Bcl-2）蛋白家族成员和它们调控的凋亡通路是肿瘤治疗非常有前景的药物靶点[1]–[2]。AbbVie和Genentech公司合作研发的venetoclax是第一个靶向Bcl-2的选择性抑制剂，2018年被美国食品药品监督管理局（FDA）批准用于治疗老年或不能耐受强化疗的急性髓系白血病（AML）患者。其研发和应用在很大程度上改善了AML患者的预后和生活质量[3]–[4]。为使venetoclax获得更好的推广运用，使更多的AML患者获益，我们就venetoclax在AML中的应用进行综述。

一、概况

venetoclax（ABT-199）是一种口服的Bcl-2选择性抑制剂，是唯BH3蛋白（Bcl-2蛋白家族成员）的类似物，通过与Bcl-2蛋白结合，替换Bim等前凋亡蛋白，触发线粒体外膜通透性改变和半胱氨酸蛋白酶的激活，促进内在凋亡途径活化导致细胞死亡。临床前和临床研究均证实venetoclax单药或联合其他化疗和去甲基化药物（HMA）可诱导AML肿瘤细胞凋亡，且患者耐受性良好。

venetoclax有10、50、100 mg三种剂型。口服给药，切勿咀嚼、压碎或打碎药片。药物口服吸收缓慢，在400 mg剂量下，在5～8 h后达到最大血浆浓度，平均C_max_为（2.1±1.1）mg /ml，AUC_0-24_为（32.8±16.9）mg·h^−1^·ml^−1^。建议在进食后半小时内服用。其主要由肝CYP3A4/5系统代谢，主要通过粪便排出（>99.9％），半衰期约为26 h。主要的不良反应为胃肠道反应，包括恶心、呕吐和腹泻。治疗过程中需警惕肿瘤溶解综合征（Tumor lysis syndrome，TLS）以及骨髓抑制。联合用药方面，一般建议与中度CYP3A4抑制剂联合使用时venetoclax剂量降低50％，与强效CYP3A4抑制剂联合使用时至少降低75％。

二、TLS的预防

由于Bcl-2抑制剂治疗初期可能导致白血病细胞快速凋亡，而诱发TLS。高乳酸脱氢酶、高白细胞计数、高尿酸、肾功能不全等因素会使TLS的风险增加[5]。尽管临床研究报道中AML的TLS发生率较慢性淋巴细胞白血病/小淋巴细胞淋巴瘤要低，但也需引起临床医师的足够重视[6]–[8]。治疗初期密切的监测和适当的预防措施可有效降低TLS的发生[9]。在venetoclax治疗开始前，建议采用羟基脲等药物将WBC降低至25×10^9^/L以下，剂量增加期间患者需要充分的静脉及口服水化碱化。从低剂量开始，每天缓慢增加剂量直至目标治疗剂量。通常，患者可以在周期的第1天以100 mg的剂量服用venetoclax，此后每天可以将剂量翻倍，直至达到目标剂量，venetoclax与HMA联合日剂量递增为100→200→400 mg，与低剂量阿糖胞苷（LDAC）联合日剂量递增为100→200→400→600 mg。剂量增加期间需要每天监测TLS相关的实验室指标（包括钾、尿酸、磷、钙和肌酐），高危患者建议频率增加至每6～8 h 1次。

三、Bcl-2抑制剂的治疗方案

1. Bcl-2抑制剂单药：venetoclax单药治疗的临床前及临床研究，证明了该药对复发/难治或不适合强化疗AML患者的耐受性和安全性。在一项复发难治及不耐受强化疗的AML患者接受venetoclax单药治疗的Ⅱ期单臂研究中，共入组32例患者，平均年龄71岁，41％的患者接受过三线以上治疗，62％的患者存在复杂或del（7q）染色体异常。患者接受每日剂量爬坡至目标剂量800 mg，多数患者起效时间为4周。19％的患者获得完全缓解（CR）或血小板和中性粒细胞均未恢复的CR（CRi），19％的患者骨髓达到部分缓解（PR），中位生存时间4.7个月。既往接受过HMA治疗的患者CR+CRi率达25％，伴有IDH突变的患者CR+CRi率为33％。主要的治疗相关不良事件为骨髓抑制，3～4级发热性中性粒细胞减少约占30％，无TLS发生，药物耐受性良好[10]。单药治疗有效但维持时间短暂，该研究也进一步推动了venetoclax与AML其他治疗药物联合的研究。

2. Bcl-2抑制剂联合HMA/LDAC：阿扎胞苷、地西他滨等HMA在老年患者中显示出了较好的疗效，但单药临床缓解率低，长期生存并不理想。venetoclax与HMA/LDAC的联合使得老年AML患者，尤其是合并症多、对化疗耐受性差的老年患者的生存显著改善。一项采用venetoclax联合LDAC治疗不耐受化疗的老年初治AML患者的Ⅰb/Ⅱ期临床研究中，82例患者中位年龄74岁，49％为继发性AML，29％曾接受过HMA治疗，32％存在预后不良细胞遗传学改变。患者每日口服600 mg目标剂量venetoclax联合LDAC（20 mg/m^2^，第1～10天）皮下注射，28 d为1个治疗周期。结果显示总体CR+CRi率达54％，中位总生存（OS）期为10.1个月。初治患者、中危核型患者及既往未接受HMA治疗患者CR+CRi率分别为71％、63％、62％。伴有NPM1、IDH突变患者的CR/CRi率分别为89％和72％，疗效高于伴有TP53、FLT3突变的患者（30％和44％）。证实了联合治疗缓解率高，不良反应发生率低，且缓解快速和相对持久[7]。另一项venetoclax联合HMA（阿扎胞苷或地西他滨）治疗不能耐受强化疗的老年初治AML的Ⅰb期研究，145例患者中位年龄74岁，25％为继发性，49％患者存在预后不良细胞遗传学改变，超过一半的患者存在TP53、FLT3、IDH或NPM1突变。所有患者接受每日口服400、800或1200 mg的venetoclax联合标准剂量地西他滨（20 mg/m^2^，第1～5天，静脉给药）或阿扎胞苷（75 mg/m^2^，第1～7天，静脉或皮下给药）28 d为1个周期的治疗。其中60例患者在venetoclax 400 mg剂量组（29例为联合阿扎胞苷组，31例为联合地西他滨组），74例患者在800 mg剂量组（每组各37例），11例患者在1200 mg剂量组（6例为联合阿扎胞苷组，5例为联合地西他滨组）。结果提示总体患者有效率（CR+CRi）为67％，伴突变阳性患者的CR/CRi率分别为：IDH 71％、NPM1 91％、FLT3 72％、TP53 47％。研究总体中位疗效持续时间为11.3个月，中位OS时间为17.5个月，其中venetoclax 400 mg联合组中位OS时间均未达到。老年患者显示出可耐受的安全性和良好的疗效[8]。近期一项阿扎胞苷±venetoclax的Ⅲ期随机试验，共纳入了431例初发老年患者，2∶1随机分配至治疗组与对照组，主要终点为OS期，venetoclax每日400 mg，阿扎胞苷75 mg/m^2^，第1～7天。结果显示，两组CR率分别为36.7％和17.9％（*P*<0.001），CR+CRi率分别为66.4％和28.3％（*P*<0.001），在20.5个月的中位随访中，联合组的中位OS时间为14.7个月，而对照组为9.6个月[11]。针对特殊突变类型的亚组研究发现伴NPM1、IDH突变者对venetoclax最敏感[12]–[13]。

3. Bcl-2抑制剂联合小分子靶向药物：小分子药物与特定的靶向药物联合治疗AML可进一步提高临床疗效。2017年至今，有8个新药获得美国FDA批准，分别为FLT3抑制剂midostaurin和gilteritinib，IDH抑制剂ivosidenib和enasidenib，CD33单抗gemtuzumab ozogamicin，脂质体包裹柔红霉素和阿糖胞苷混合药物CPX351以及glasdegib和Bcl-2抑制剂venetoclax[14]。目前Bcl-2抑制剂与FLT3抑制剂以及IDH1/IDH2抑制剂的联合正在进行临床研究。

临床上20％～30％的AML患者合并FLT3-ITD突变，目前有多种FLT3的小分子靶向抑制药物[15]。研究发现FLT3抑制剂与venetoclax联合可有效提高FLT3抑制剂的作用[16]。这可能与FLT3抑制剂可有效减少FLT3阳性细胞的MCL-1转入和保持MCL-1蛋白稳定性相关。目前有数个venetoclax与FLT3抑制剂联合的临床试验正在进行中。IDH 1/2突变在AML中的发生率为7％～15％，老年患者中比例更高[17]。IDH1抑制剂（ivosidenib）和IDH2抑制剂（enasidenib）与venetoclax的联合也是目前临床研究的重点。有研究提出IDH1和IDH2突变可通过2-HG介导的细胞色素C氧化酶活性的降低来降低诱导凋亡的线粒体阈值，从而提高对Bcl-2抑制的敏感性[18]。

4. Bcl-2抑制剂联合标准化疗：对于没有特定靶点突变但适合强烈化疗的老年患者，采用venetoclax联合标准诱导方案将是一个不错的治疗选择。研究表明，与强化疗的联合在增加疗效的同时可以降低venetoclax耐药的发生。目前已有数篇相关的研究报道。在一项针对复发/难治性AML的临床试验中，13例接受FLA-IDA联合venetoclax（FLAVIDA）方案（氟达拉滨、阿糖胞苷、G-CSF和去甲氧柔红霉素联合venetoclax 100 mg第1～7天,考虑到患者须采用CYP3A4抑制剂预防真菌感染）的患者，与既往81例相同入组标准的采用FLA-IDA挽救治疗的患者进行比较。1个疗程FLAVIDA方案治疗后的总缓解率（ORR）为69％，中位CR持续时间为7.3个月，显著好于FLA-IDA对照组的47％，FLAVIDA组9例ORR患者中2例微小残留病（MRD）转阴性，为后续的异基因造血干细胞移植提供了机会。研究结果提示短期的venetoclax联合强烈挽救化疗有效且不良反应安全可控[19]。另一项针对老年可耐受强化疗AML患者的研究采用了venetoclax（50～600 mg剂量爬坡，−6 d至+7 d）联合IA（阿糖胞苷100 mg/m^2^，第1～5天；去甲氧柔红霉素12 mg/m^2^，第1～2天）的诱导方案，后续予以venetoclax联合化疗的巩固治疗。51例患者中位年龄72岁，总体CR/CRi率高达72％，初发患者CR/CRi率高达97％，继发性AML患者CR/CRi率43％，且患者耐受性良好，主要3级及以上的非血液学不良反应为粒细胞缺乏性发热（55％）和脓毒血症（35％）[20]。联合用药同样提示NPM1和IDH1/2突变亚组的疗效最好。

5. Bcl-2抑制剂耐药与对策：关于Bcl-2耐药机制目前仍不明确，可能与多种因素相关。venetoclax的耐药性可能与Bcl-2家族蛋白MCL-1以及Bcl-X_L_的上调相关[21]–[22]。Nechiporuk等[23]采用CRISPR/Cas9基因编辑技术分别敲除了TP53、BAX和PMAIP1基因，以探究venetoclax在AML患者中的耐药机制。研究结果提示TP53凋亡网络是venetoclax在AML治疗中耐药的关键因素。DiNardo等[24]分析了81例采用venetoclax为基础的联合化疗的老年AML患者，根据治疗反应，患者被分为4组：持续缓解组、缓解后复发组（适应性耐药）、原发性难治性组、未分类组，以探究各组的分子相关性。得出两个重要结论，适应性耐药可能是多克隆的，并与TP53异常或激酶激活有关，特别是和FLT3-ITD相关。持续缓解组中，NPM1突变与联合化疗后的良好的生存前景和持久的分子缓解相关。

药物联合可能克服venetoclax的耐药。研究提示靶向MCL-1和Bcl-X_L_可使患者重获对venetoclax的敏感性，venetoclax与MCL-1或BCL-X_L_抑制剂早期联合也可以延缓耐药的发生。由于Bcl-X_L_抑制剂导致血小板减少及其他不良反应，MCL-1抑制剂可能更具有临床应用价值。MCL-1的下调也可以通过干扰MAPK、p53和CDK9途径来间接实现，目前MEK抑制剂、E3泛素蛋白连接酶MDM2（p53的负调节剂）、CDK9抑制剂与venetoclax联合的临床前和临床研究也在进行中。

四、小结

venetoclax联合HMA或LDAC在AML治疗中具有里程碑意义，为不适合强化疗甚至适合强化疗的老年患者提供了新的一线治疗选择。特殊突变（IDH1、IDH2和NPM1）对Bcl-2抑制剂联合方案尤其敏感。对于可以耐受强烈化疗的年轻患者，采用venetoclax联合标准化疗方案可以带来获益。venetoclax还为具有特殊突变（如FLT3、IDH）的患者采用靶向药物的联合治疗提供了可能。下一步的研究重点将是确定venetoclax最佳剂量以及联合治疗方案。

## References

[1] Juárez-Salcedo LM, Desai V, Dalia S (2019). Venetoclax: evidence to date and clinical potential[J]. Drugs Context.

[2] Lachowiez C, DiNardo CD, Konopleva M (2020). Venetoclax in acute myeloid leukemia - current and future directions[J]. Leuk Lymphoma.

[3] Guerra VA, DiNardo C, Konopleva M (2019). Venetoclax-based therapies for acute myeloid leukemia[J]. Best Pract Res Clin Haematol.

[4] EDV C, Pinto R (2019). Targeted therapy with a selective BCL-2 inhibitor in older patients with acute myeloid leukemia[J]. Hematol Transfus Cell Ther.

[5] Montesinos P, Lorenzo I, Martín G (2008). Tumor lysis syndrome in patients with acute myeloid leukemia: identification of risk factors and development of a predictive model[J]. Haematologica.

[6] Roberts AW, Davids MS, Pagel JM (2016). Targeting BCL2 with venetoclax in relapsed chronic lymphocytic leukemia[J]. N Engl J Med.

[7] Wei AH, Strickland SA, Hou JZ (2019). Venetoclax combined with low-dose cytarabine for previously untreated patients with acute myeloid leukemia: results from a phase Ib/II study[J]. J Clin Oncol.

[8] DiNardo CD, Pratz K, Pullarkat V (2019). Venetoclax combined with decitabine or azacitidine in treatment-naive, elderly patients with acute myeloid leukemia[J]. Blood.

[9] Richard-Carpentier G, DiNardo CD (2019). Venetoclax for the treatment of newly diagnosed acute myeloid leukemia in patients who are ineligible for intensive chemotherapy[J]. Ther Adv Hematol.

[10] Konopleva M, Pollyea DA, Potluri J (2016). Efficacy and biological correlates of response in a phase II study of venetoclax monotherapy in patients with acute myelogenous leukemia[J]. Cancer Discov.

[11] DiNardo CD, Jonas BA, Pullarkat V (2020). Azacitidine and venetoclax in previously untreated acute myeloid leukemia[J]. N Engl J Med.

[12] Lachowiez CA, Loghavi S, Kadia TM (2020). Outcomes of older patients with NPM1-mutated AML: current treatments and the promise of venetoclax-based regimens[J]. Blood Adv.

[13] Tiong IS, Dillon R, Ivey A (2021). Venetoclax induces rapid elimination of NPM1 mutant measurable residual disease in combination with low-intensity chemotherapy in acute myeloid leukaemia[J]. Br J Haematol.

[14] Rowe JM (2019). Will new agents impact survival in AML?[J]. Best Pract Res Clin Haematol.

[15] Daver N, Schlenk RF, Russell NH (2019). Targeting FLT3 mutations in AML: review of current knowledge and evidence[J]. Leukemia.

[16] Aldoss I, Zhang J, Mei M (2020). Venetoclax and hypomethylating agents in FLT3-mutated acute myeloid leukemia[J]. Am J Hematol.

[17] Ley TJ, Miller C, Ding L (2013). Genomic and epigenomic landscapes of adult de novo acute myeloid leukemia[J]. N Engl J Med.

[18] Chan SM, Thomas D, Corces-Zimmerman MR (2015). Isocitrate dehydrogenase 1 and 2 mutations induce BCL-2 dependence in acute myeloid leukemia[J]. Nat Med.

[19] Shahswar R, Beutel G, Klement P (2020). FLA-IDA salvage chemotherapy combined with a seven-day course of venetoclax (FLAVIDA) in patients with relapsed/refractory acute leukaemia[J]. Br J Haematol.

[20] Chua CC, Roberts AW, Reynolds J (2020). Chemotherapy and venetoclax in elderly acute myeloid leukemia trial (CAVEAT): a phase Ib dose-escalation study of venetoclax combined with modified intensive chemotherapy[J]. J Clin Oncol.

[21] Niu X, Zhao J, Ma J (2016). Binding of released bim to mcl-1 is a mechanism of intrinsic resistance to ABT-199 which can be overcome by combination with daunorubicin or cytarabine in AML cells[J]. Clin Cancer Res.

[22] Lin KH, Winter PS, Xie A (2016). Targeting MCL-1/BCL-XL forestalls the acquisition of resistance to ABT-199 in acute myeloid leukemia[J]. Sci Rep.

[23] Nechiporuk T, Kurtz SE, Nikolova O (2019). The TP53 apoptotic network is a primary mediator of resistance to BCL2 inhibition in AML Cells[J]. Cancer Discov.

[24] DiNardo CD, Tiong IS, Quaglieri A (2020). Molecular patterns of response and treatment failure after frontline venetoclax combinations in older patients with AML[J]. Blood.

